# Effect of protracted dexamethasone exposure and its withdrawal on rocuronium-induced neuromuscular blockade and sugammadex reversal: an *ex vivo* rat study

**DOI:** 10.1038/s41598-019-47784-3

**Published:** 2019-08-02

**Authors:** Seok Kyeong Oh, Byung Gun Lim, Sungsoo Park, Hong Seuk Yang, Junyong In, Yong Beom Kim, Hey-ran Choi, Il Ok Lee

**Affiliations:** 1 Department of Anaesthesiology and Pain Medicine, Korea University Guro Hospital, Korea University College of Medicine, Seoul, Republic of Korea; 2 Department of Surgery, Korea University Anam Hospital, Korea University College of Medicine, Seoul, Republic of Korea; 3 Department of Anaesthesiology and Pain Medicine, Asan Medical Center, Seoul, Republic of Korea; 4 Department of Anaesthesiology and Pain Medicine, Dongguk University Ilsan Hospital, Goyang, Republic of Korea; 5 Department of Anaesthesiology and Pain Medicine, Gachon University Gil Hospital, Incheon, Republic of Korea; 6 Department of Anaesthesiology and Pain Medicine, Inje University Seoul Paik Hospital, Seoul, Republic of Korea

**Keywords:** Neurophysiology, Preclinical research

## Abstract

Studies have reported that protracted dexamethasone treatment induces resistance to nondepolarizing neuromuscular blocking agents (NMBAs) and the association with nicotinic acetylcholine receptors in the diaphragm of rats. Here, we investigated the effect of protracted dexamethasone administration on the sensitivity to rocuronium and the recovery profile when reversed by sugammadex; additionally, we observed the recovery period of pharmacodynamic change after withdrawal. Sprague-Dawley rats received daily intraperitoneal injections of dexamethasone or saline for 14 days. On days 1, 3, and 7 after the last dexamethasone treatment (Dexa1, Dexa3, and Dexa7, respectively) or 1 day after saline (control group), the phrenic nerve-hemidiaphragm preparation was dissected for assay. The dose-response curve of rocuronium in Dexa1 was shifted to the right compared to controls, but curves in Dexa3 and Dexa7 were not significantly different. Groups were not significantly different in attaining the train-of-four ratio ≥ 0.9, but the recovery index in Dexa7 was shorter than that in control and Dexa1. Recovery profiles (period of sugammadex reversal) were not correlated with resistance properties but rather with total administered drugs (binding capacity of NMBAs and sugammadex). Protracted dexamethasone exposure induced resistance to rocuronium but seemed to have no effect on sugammadex reversal in the rat diaphragm.

## Introduction

Synthetic glucocorticoids are used to control inflammatory and autoimmune diseases, in the treatment of certain types of leukaemia, and for achieving immunosuppression after organ transplant. However, their clinical usefulness is compromised by the side effects of their long-term use; potential side effects include osteoporosis, hypertension, dyslipidaemia, insulin resistance/type 2 diabetes mellitus, increased intraocular pressure, and adrenal insufficiency^1,2^. With regard to the neuromusculature, long-term treatment with glucocorticoids (including dexamethasone) has been reported to induce muscle atrophy, dysfunction^3–5^ and resistance to nondepolarizing neuromuscular blocking agents (NMBAs)^6,7^.

Long-term glucocorticoid-induced hyposensitivity to numerous nondepolarizing NMBAs such as d-tubocurarine,atracurium, vecuronium, and rocuronium has been observed in a number of studies^6,8–12^. Glucocorticoids induce resistance to NMBAs by increasing the surface nicotinic acetylcholine receptor density (nAChRs; as seen with dexamethasone)^13^. Additionally, long-term treatment with glucocorticoids increases the accumulation of junctional nAChRs because of decreased degradation and increased synthesis of nAChRs^14^. Protracted dexamethasone treatment induces upregulation of nAChRs in a concentration-dependent manner^15^ and results in alteration of immature form (γ-subunit) nAChRs in the diaphragms of rats^6^. AChRs are upregulated with the increased immature isoform in some pathologic states such as denervation, stroke, sepsis, burns, immobilization, long-term use of nondepolarizing NMBAs^16–18^, and protracted corticosteroid use^10,11^. Recently, the presence of another isoform of the nAChRs, the α7-nAChR, previously described only in the neuronal tissues (brain), has been identified in the muscle tissues during immobilization, sepsis, and denervation^17,19,20^. The α7-nAChRs differ from conventional muscle nAChRs in their functional and pharmacologic traits, and these altered functional and pharmacologic properties of the neuronal α7-nAChRs confer resistance to nondepolarizing NMBAs^17^.

Muscle atrophy caused by dexamethasone administration, and the subsequent recovery of muscle mass after withdrawal from dexamethasone treatment have been examined in a previous study^5^. This study measured the leucine responsiveness of muscle protein synthesis in young rats (4–5 weeks of age) treated with dexamethasone and reported partial and near recovery of muscle protein synthesis, 3 and 7 days, respectively, after withdrawal of dexamethasone treatment.

The effect of protracted exposure to dexamethasone on sugammadex reversal has not been investigated, and nor has the consequent recovery of the dexamethasone-induced neuromuscular pharmacodynamic change after discontinuation of dexamethasone treatment. This dearth of research directed the two objectives of the current study. The first objective was to investigate the effect of protracted dexamethasone administration on the sensitivity to rocuronium-induced neuromuscular block (blockade) and on the recovery profile when the blockade was reversed by sugammadex (reversal). The second was to observe the recovery period of pharmacodynamic change to normal level after discontinuation of dexamethasone administration. We hypothesized that the dexamethasone-treated group would show more resistance to NMBAs than the control group, and faster reversal by sugammadex due to up-regulation of the immature nAChRs. Moreover, we hypothesized that the recovery period would last 3–7 days, with the recovery period defined as adequate time to recover the normal pharmacodynamic effects of NMBAs and sugammadex as comparable to a previous study^5^. Hence, we investigated the function of the rat diaphragm *ex vivo* in relation to the sensitivity to rocuronium, with a cumulative dose-response method, and observed the recovery profiles after sugammadex administration. Additionally, we also assessed the neuromuscular properties in association with the expression of α7-nAChRs.

## Results

### Animal and specimen data

Initially, 18 rats were assigned to each group. Two rats in the Dexa1 group died during the treatment period of 14 days. The cause of death might have been the side effects of dexamethasone, which manifested as reduced food intake, consequent decline in body weight, muscle weakness, and dexamethasone-induced metabolic changes. Some phrenic nerve-hemidiaphragm preparations were not functioning well or not fully recovered during the *ex vivo* experiment, therefore they were excluded from the analysis. In total, 63 rats (17 in the control group, 14 in Dexa1, 16 in Dexa3, and 16 in Dexa7) were included in the analyses.

There was no significant intergroup difference in the initial body weight before the experimental treatment, but the final body weight after the experimental treatment significantly differed between the groups. Food intake decreased markedly among the rats during the dexamethasone treatment period^4–7,21^. The size and weight of *ex vivo* preparations were not different between groups (Table 1).

**Table 1:** Animal and specimen data.

	Control	Dexa1	Dexa3	Dexa7	P-value
**Effect of experimental treatment on body weight**					
Initial Body Weight (g)	241.0 (4.6)	232.2 (20.8)	236.9 (19.6)	234.5 (20.2)	0.543
Final Body Weight (g)	374.6 (19.6)^#▴◆^	227.8 (24.0)	239.6 (28.7)	275.7 (19.0)^#▴^	<0.001
**Size and weight of specimens**					
Length (mm)	14.64 (1.86)	13.42 (1.45)	14.00 (1.66)	13.93 (1.48)	0.276
Width (mm)	12.71 (1.32)	12.00 (1.75)	11.28 (1.20)	11.94 (1.51)	0.090
Weight of diaphragm (mg)	185 (25)	153 (32)	168 (43)	185 (38)	0.061
**Weight of Total specimen (mg)**					
(diaphragm + thoracic wall)	891 (125)	752 (118)	819 (155)	861 (172)	0.077

Values are mean (SD). Statistical analysis was carried out by one-way analysis of variance with the *post hoc* Bonferroni test, *P < 0.05 vs the control group; ^#^P < 0.05 vs the Dexa1 group; ^▴^P < 0.05 vs the Dexa3 group; ^◆^P < 0.05 vs the Dexa7 group.

### Rocuronium dose-response curves

**Figure 1:**
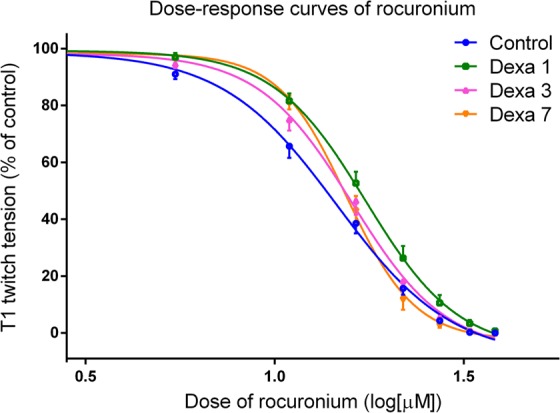
Dose-response curves of rocuronium in the Dexa1, Dexa3, Dexa7, and control groups. The x-axis represents the logarithm of the rocuronium concentration. Each point represents the mean ± SEM of the percent twitch tension inhibition. The dose–response curves for rocuronium were significantly different between experimental groups (P < 0.05, each by Bonferroni test). The dose–response curves of the Dexa1 group were significantly different and shifted to the right as compared to those of the control group (P < 0.05). The curves of the other groups were not significantly different (P > 0.05).

Rocuronium reduced the indirectly-elicited twitch tension in a dose-dependent manner in all groups. The dose-response curves of the Dexa1 group were significantly shifted to the right as compared with those of the control group (P = 0.023). No significant difference was found among the curves of the other groups (P > 0.05; Fig. 1).

The 50% inhibitory concentration (IC50) value, which quantitatively describes the position on the curve, was significantly increased in Dexa1 group (P = 0.027). The slope at IC50 was significantly steeper in Dexa7 group than in the other groups (P < 0.05; Table 2).

**Table 2:** IC50 values (µM) and slopes of the rocuronium dose-response curves.

	Control	Dexa1	Dexa3	Dexa7
IC50	14.62 (13.66–15.65)	17.42* (16.31–18.61)	16.02 (15.05–17.06)	15.77 (15.10–16.47)
LogIC50	1.165 (1.135–1.194)	1.241* (1.212–1.270)	1.205 (1.178–1.232)	1.198 (1.179–1.217)
Slope at IC50	−2.867 (0.303)	−3.550 (0.170)	−3.499 (0.366)	−4.905*^#▴^ (0.410)
IC50 ratio		1.197 (1.138–1.256)	1.092 (1.039–1.145)	1.081 (1.028–1.133)

IC50 = 50% inhibitory dosage. IC50 ratio = (IC50 of the Dexa group dose–response curves/IC50 of the control group dose–response curve).Values are mean (95% confidence intervals) and mean (SEM). *P < 0.05 vs the control group; ^#^P < 0.05 vs the Dexa1 group; ^▴^P < 0.05 vs the Dexa3 group.

The total molar concentrations of administered rocuronium in the organ bath were 27.0 ± 2.3, 30.8 ± 4.0, 27.3 ± 4.4, and 24.9 ± 3.9 (mean ± SD µM) in the control, Dexa1, Dexa3, and Dexa7 groups, respectively. The molar concentrationof total sugammadex administered in the organ bath was double the molar concentration of total rocuronium administered.

### Recovery profiles

As the primary outcome, the time from administration of sugammadex to a train-of-four (TOF) ratio of at least 0.9 (time to TOF ratio ≥ 0.9) was not significantly different between the groups. However, the recovery index (time taken for recovery of the first twitch from 25% to 75%) and the time for recovery of T1 (height of the first twitch) to 95% in Dexa7 group were significantly shorter than those in the control and Dexa1 groups (P < 0.05; Table 3).

**Table 3:** Recovery time after sugammadex administration.

	Control	Dexa1	Dexa3	Dexa7
Time to TOF ratio ≥ 0.9	12.8 (10.2–15.4)	15.0 (10.9–19.1)	10.2 (8.4–12.0)	10.1 (8.1–12.1)
Recovery index	5.4 (4.4–6.3)	5.8 (3.9–7.7)	3.8 (3.1–4.5)	3.1*^#^ (2.5–3.7)
Time of T1 recovery to 95%	11.6 (9.2–13.9)	11.5 (9.1–14.0)	9.7 (7.9–11.5)	7.4*^#^ (6.2–8.6)

(min) Values are mean (95% confidence intervals). T1: height of the first twitch. *P < 0.05 vs the control group; ^#^P < 0.01 vs the Dexa1 group.

Time to TOF ratio ≥ 0.9 and recovery index were 12.8 ± 4.9, 15.0 ± 7.1, 10.2 ± 3.3, and 10.1 ± 3.8 and 5.4 ± 1.9, 5.8 ± 3.3, 3.8 ± 1.3, and 3.12 ± 1.2 (mean ± SD min) in the control,Dexa1, Dexa3, and Dexa7 groups, respectively (Table 3).

### Histopathological examination

**Figure 2:**
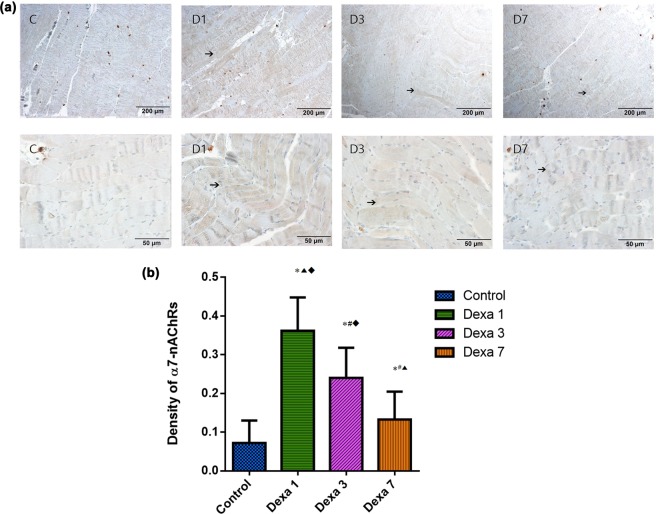
(**a**) Immunohistochemical staining of α7- nicotinic acetylcholine receptors (nAChRs) in the diaphragm muscle samples. The expression of α7-nAChRs was detected on the membrane of skeletal muscle cells and appeared in the all Dexa groups, peaking in the Dexa1 group and declining gradually in the Dexa3 and Dexa7 groups. C = control, D1 = Dexa1, D3 = Dexa3, D7 = Dexa7. (**b**) Average optical density of α7-nAChRs in the different treatment groups. All values are expressed as mean ± SD (n = 6). *P < 0.05 vs the control group; ^#^P < 0.05 vs the Dexa1 group; ^▴^P < 0.05 vs the Dexa3 group; ^◆^P < 0.05 vs the Dexa7 group.

The expression of α7-nAChRs was detected on the membrane of skeletal muscle cells; expression appeared in all dexamethasone-treated groups, peaking in the Dexa1 and declining gradually in the Dexa3 and Dexa7 groups. In the control group, α7-nAChRs was scarcely observed (Fig. 2).

The results of average optical density were 0.072 ± 0.058, 0.362 ± 0.086, 0.240 ± 0.078, and 0.133 ± 0.072 (mean ± SD) in the control, Dexa1, Dexa3, and Dexa7 groups, respectively.

### Correlation analysis

The total molar concentrations of administered rocuronium were positively correlated with the IC50 (r = 0.735, P < 0.001).The level of α7-nAchRs expression and the IC50 of rocuronium were positively statistically correlated (r = 0.555, P = 0.005), as were the α7-nAchRs expression and the total molar concentrations of administered rocuronium in the organ bath (r = 0.534, P = 0.007).

**Figure 3:**
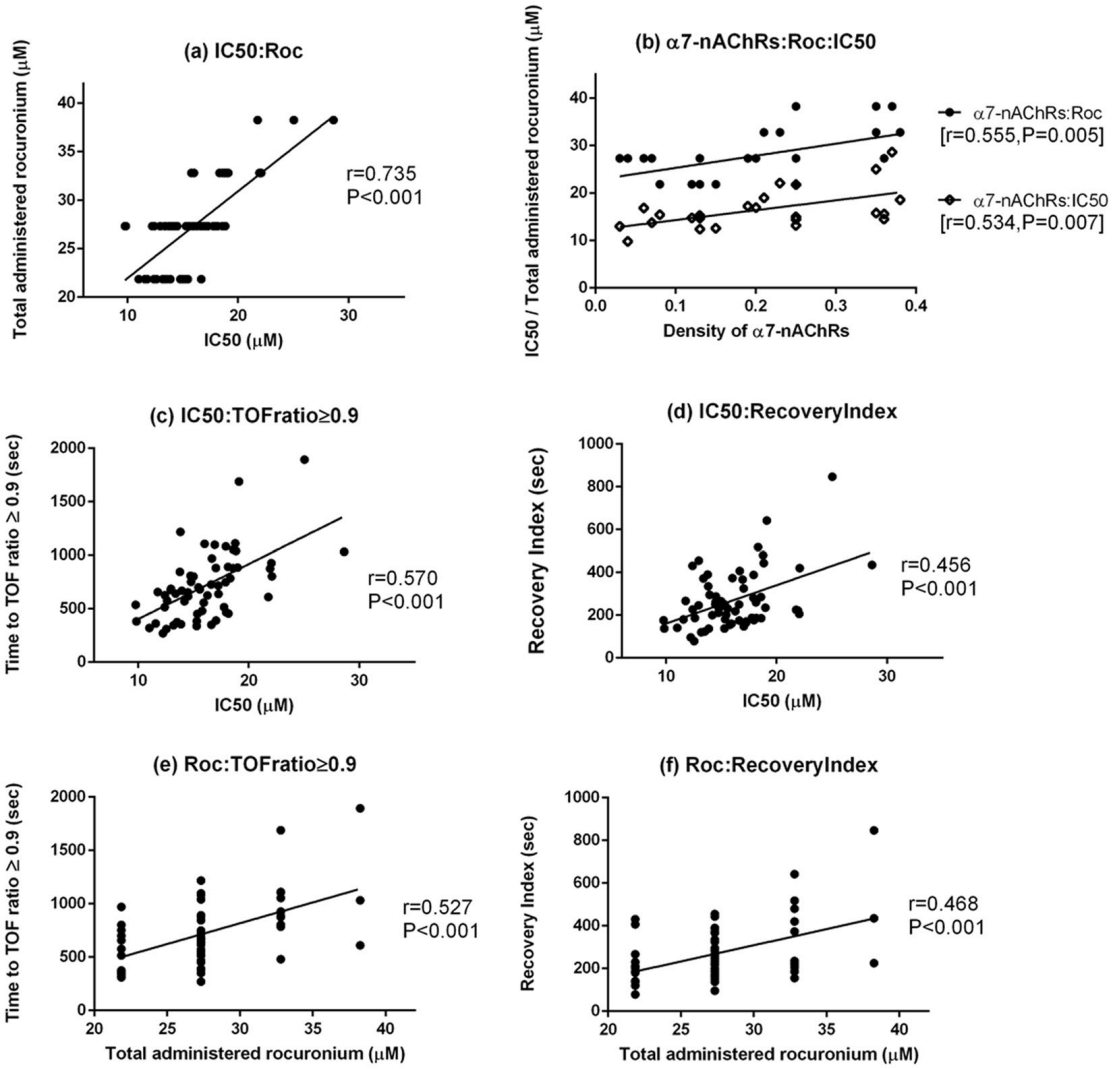
Correlation analysis of variables in association with recovery profiles. Values are Pearson’s coefficients of correlation. IC50 = 50% inhibitory concentration. Roc = the total rocuronium administered into the organ bath in molar concentration (µM of total administered rocuronium = half µM of total administered sugammadex). α7-nAChRs = The average optical density of α7- nicotinic acetylcholine receptors. TOF ratio ≥ 0.9 = Time to TOF ratio ≥ 0.9. Recovery Index = recovery index (time to recovery of the first twitch from 25% to 75%). (**a**) The correlation of IC50 with Roc. (**b**) The correlation of the average optical density of α7 nAChRs with IC50 and Roc. (**c**) The correlation of IC50 with the time to TOF ratio ≥ 0.9. (**d**) The correlation of IC50 with recovery index. (**e**) The correlation of Roc with the time to TOF ratio ≥ 0.9. (**f**) The correlation of Roc with recovery index.

Recovery profiles were positively correlated with the IC50 value (time to TOF ratio ≥ 0.9 and IC50: r = 0.570, P < 0.001; recovery index and IC50: r = 0.456, P < 0.001). Recovery profiles were also significantly positively correlated with the total administered rocuronium (time to TOF ratio ≥ 0.9 and total administered rocuronium: r = 0.527, P < 0.001; recovery index and total administered rocuronium: r = 0.468, P < 0.001; Fig. 3).

## Discussion

The results of this study confirm that long-term dexamethasone treatment inducesresistance to nondepolarizing NMBAs^6,7^; this study is the first to demonstrate the effect of long-term dexamethasone treatment on sugammadex reversal, and the association of its neuromuscular pharmacodynamics with altered α7-nAChRs expression. Additionally, this study is the first to profile the neuromuscular recovery to normal physiological response levels after withdrawal of dexamethasone treatment.

The relationship between resistance to nondepolarizing NMBAs in dexamethasone-treated rat diaphragm and altered expression of the γ–subunits of nAChRs has been previously reported by Chen *et al*.^6^. The alteration in the subunit composition (γ/ε) of nAChRs confers certain changes in functional, pharmacologic, and metabolic responses^17,18,22^. The γ–subunit may also change the sensitivity or affinity of the receptor for specific ligands, thereby developing resistance to nondepolarizing NMBAs. Although the association of altered γ-nAChRs expression and protracted dexamethasone treatment has been explored previously, our study is the first to examine whether α7-nAChRs expression also occurs after protracted dexamethasone treatment. According to a study by Lee *et al*.^17^, resistance to nondepolarizing NMBAs, occurring after immobilization, might be related not with the immature nAChRs, but with up-regulated α7-nAChRs. In this regard, α7-nAChRs may play a pivotal role in conferring resistance to NMBAs; hence, further investigation of the relationship between dexamethasone-induced upregulated α7-nAChRs and resistance to nondepolarizing NMBAs is essential to determine the mechanism of this resistance.

IC50 represents the concentration of a drug that is required for 50% inhibition, and is used as a measure of drug potency. In this study, the IC50 values were used as measures of the pharmacodynamic response (resistance) to rocuronium, as in the previous studies^23,24^. As measured by the IC50, resistance to rocuronium was increased in Dexa1 group and decreased in Dexa3 and Dexa7 groups; these results were well correlated with the expression of α7 receptors assessed by immunohistochemistry. However, the slope at IC50 was significantly steeper in Dexa7 group. This difference in the Dexa7 group may have two possible explanations. One, difference in the age of the experimental rats in Dexa7 group; rats in this group were one week older than those in the control and Dexa1 groups, and 4 days older than Dexa3 group. The 7-week-old rats were treated for 2 weeks; hence, the diaphragms harvested from 9–10-week-old rats were used in this study. The rat age of 9–10 weeks is in the period from peri-adolescence (7 weeks) to young adulthood (10 weeks). For a rat in adolescence and young adulthood, one day is approximately equivalent to 34.8 human days^25^; therefore, difference of 4 and 7 days in the age of rats might not be negligible, especially for a rat in growth phase. Second possible explanation is the change in sensitivity (enhancement) after dexamethasone withdrawal by an unknown mechanism. This kind of enhanced response to acetylcholine after discontinuing corticosteroid treatment with was reported by a previous study, though in a different animal model (dog) and in a different target organ (airway)^26^. The mechanism of this enhancement has not been discovered yet but may be due to an abrupt withdrawal of corticosteroid treatment. Since the presence of nAChRs has been reported in the airway epithelium^27,28^, a similar phenomenon could have occurred at the nAChRs in the neuromuscular junction. To our knowledge, the pharmacodynamic change after withdrawal from long-term dexamethasone treatment has never been explored previously; therefore, further study may be needed to identify this phenomenon.

Only a few *in vitro* or *ex vivo* studies exist regarding the interaction of rocuronium and sugammadex^29–33^; thus, there is no consensus on the recommended dose of sugammadex to be used in an *ex vivo* study. Previously, we have used an equimolar dose of sugammadex and rocuronium^30,33^; this was based on the mechanism of action of sugammadex which encapsulates the free rocuronium molecules in 1: 1 molecular ratio^34^. However, in a clinical setting, a 0.6 mg kg^−1^ dose of rocuronium is routinely used, and 4 mg kg^−1^ dose of sugammadex is recommended by the manufacturer’s guideline when no twitch response on a TOF (and post-tetanic count ≥ 1) is seen. The molar concentration ratio of 4 mg sugammadex and 0.6 mg rocuronium is about 1.866. Furthermore, considering the diminished plasma concentration of rocuronium until the administration of sugammadex, the ratio would be increased when the two drugs are binding. Therefore, an equimolar dose of sugammadex was considered to be insufficient to reflect routine clinical practice; therefore, twice the equimolar dose of sugammadex was planned for use in this study.

Time to recovery of a TOF ratio of at least 0.9 is considered the “gold standard” for neuromuscular recovery after administration of nondepolarizing NMBAs and an NMBA antagonist^35,36^. Hence, time to TOF ratio ≥ 0.9 was selected as the primary outcome among the recovery profiles. Time to TOF ratio ≥ 0.9 was not significantly different between the groups in our study; thus, the protracted dexamethasone administration can be considered to not affect sugammadex reversal.

T1 recovery in the Dexa7 group was faster than that in the control and Dexa1 groups. These results were inconsistent with our hypothesis that the dexamethasone-treated group (especially Dexa1 group) would show faster reversal by sugammadex due to up-regulation of immature nAChRs as compared to the control group. In previous clinical experiments, a shorter duration of neuromuscular blocking was observed in the corticosteroid treated group when recovering spontaneously^10,11^. This means that the larger the resistance, the faster the recovery (smaller recovery profiles). In this regard, it was expected that recovery profiles would be inversely (negatively) correlated with IC50 (representing resistance). However, in our study the correlation of IC50 with recovery profiles was positive, as was the correlation of IC50 and the total molar concentration of administered drugs. Therefore, it is speculated that the time to sugammadex-mediated recovery from neuromuscular blockade may be correlated with the binding capacity of the NMBAs and sugammadex instead of the resistance property of receptors. This could be explained by the fact that the reversal mechanism of sugammadex is not associated with the neuromuscular junction but with the chemical-encapsulating of the amino steroidal NMBAs with high affinity^37^. When the neuromuscular block is reversed by sugammadex (unlike with spontaneous recovery or by cholinesterase inhibitors), the time to recovery from the neuromuscular block seems to be determined by the binding of the NMBAs and sugammadex rather than the up-or down-regulated nAChRs.

Nondepolarizing NMBAs are frequently used for anaesthetic management for patients who have used corticosteroids for a long period. More nondepolarizing NMBAs are required for these patients to achieve same degree of neuromuscular blockade, though the reversal of neuromuscular blockade by sugammadex may not be influenced.

There were several limitations to this study. First, only an *ex vivo* setting was employed, which means that only a pharmacodynamic model (but not a pharmacokinetic model) was evaluated. Therefore, clinical applications from our result may be limited. Nevertheless, the result from our study will provide a preliminary theoretical basis for the rational application of sugammadex in patients with protracted steroid use. Second, only young rats (around 8 weeks) were used in this study. Steroid use is more frequent in the elderly; therefore, a similar study involving older rats could be more relevant to clinical steroid use. Third, only one kind of control group (sham to Dexa1 group) was assigned. Assigning sham groups equivalent to each experimental group could have provided more information, especially regarding the unexpected difference in the Dexa7 group.

In conclusion, protracted exposure to dexamethasone causes resistance of the rat diaphragm to rocuronium as demonstrated by a right shift in dose-response curves, and the pharmacodynamic change resulting from dexamethasone treatment was restored to near normal by 3 days after treatment cessation in 7–8-week-old rats. The TOF-ratio ≥ 0.9 when reversed with sugammadex was not affected by dexamethasone administration; however, the recovery index in the group sampled 7 days after dexamethasone withdrawal was shorter than that of the control group and the group sampled 1 day after dexamethasone withdrawal. The protracted exposure to dexamethasone seemed to have no effect on sugammadex reversal in the rat diaphragm, as the reversal period by sugammadex seemed to be correlated with the binding capacity of the NMBAs and sugammadex but not with the resistance property.

## Methods

### Animals and group assignments

The study protocol was approved prior to commencing the study by the Ethics Committee of the Laboratory of Animal Research, Asan Institute for Life Science (Seoul, Republic of Korea) on February 13, 2017 (Protocol No. 2017-13-035; Chairperson Professor Jong Yeun Park), and all studies were reviewed and performed in accordance with the guidelines and regulations established by the Institutional Animal Care and Use Committee (IACUC) of Asan Institute for Life Science, Asan Medical center. The committee abides by the Institute of Laboratory Animal Resources (ILAR) guide.

**Figure 4:**
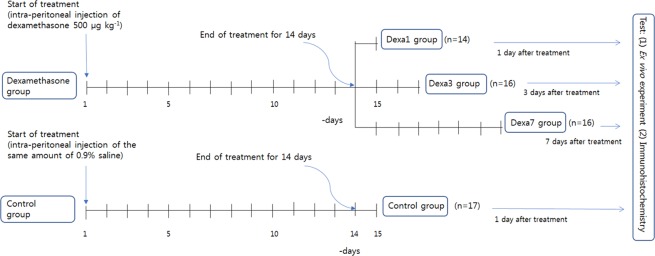
A flow diagram of the experiments.

Seventy-two male Sprague Dawley rats (7-week-old; weighing 202–271 g) were randomly assigned to dexamethasone treatment or control groups for 14 days. (Flow diagram of the experiment is shown in Fig. 4) The dexamethasone group received daily intraperitoneal injections of dexamethasone (500 μg kg^−1^ of body mass); a similar dose has been previously reported to cause muscle atrophy^6,7,38^. Dexamethasone disodium phosphate injection 5 mg ml^−1^ amp (Yuhan, Seoul, Korea) was diluted with normal saline to 500 μg ml^−1^. Therefore, for rats weighing 200–300 g, 100–150 μg of dexamethasone in a 0.2–0.3 cc volume, was injected. The control group received the same amount of saline (0.9% NaCl). The rats in the dexamethasone group were assigned to three subgroups, Dexa1, Dexa3, and Dexa7, based on the time of day for dexamethasone treatment withdrawal. For each experimental group, 18 rats were assigned. The rats were raised at a constant temperature of 22 °C and were maintained under a regular diurnal (12-h light and 12-h dark) cycle with food and water supplied *ad libitum*. This animal study complied with the ARRIVE guidelines^39^. At 1, 3, or 7 days after the last dexamethasone dose (Dexa1, Dexa3, Dexa7 group, respectively), or 1 day after normal saline (control group), the rats were anaesthetized with Alfaxan® (Jurox Pty. Limited, New South Wales, Australia) at 2 mg per 100 g of body mass intraperitoneally; if this was not effective, an additional 1 mg per 100 g was injected; rats were then sacrificed for the experiment. The left hemidiaphragm with attached phrenic nerve, central tendon, and intact rib cage were rapidly removed.

### Muscle preparations and neuromuscular pharmacodynamics

The diaphragms with phrenic nerve attached were excised *en bloc*, and the isolated phrenic nerve-hemidiaphragm was prepared. The phrenic nerve-hemidiaphragm was mounted vertically in an organ bath filled with 75 ml Krebs buffer solution (118 mmol l^−1^ NaCl, 30 mmol l^−1^ NaHCO_3_, 11.4 mmol l^−1^ glucose, 5.0 mmol l^−1^ KCl, 2.5 mmol l^−1^ CaCl_2_, 1.0 mmol l^−1^ KH_2_PO_4_ and 1.0 mmol l^−1^ MgCl_2_). The bath solution was bubbled with 95% O_2_ and 5% CO_2_ while pH and temperature were maintained at 7.4 and 35 °C, respectively. The peripheral portion of the phrenic nerve was positioned to bipolar platinum electrodes and the central tendinous portion was suspended with a force displacement transducer (Grass FT03, Grass Instrument Co., Quincy, Massachusetts, USA) using a 3–0 silk suture to measure the isometric contraction of the hemidiaphragm with a resting tension of 2 g. Using a nerve stimulator (S88, Grass) and stimulation isolation unit (SIU5, Grass), TOF stimulation (frequency 2 Hz, duration 0.2 ms) consisting of four rectangular supramaximal electrical stimuli was applied every 20 s to the phrenic nerve through the bipolar platinum electrodes. The twitch responses were recorded and displayed with a PowerLab acquisition system (ADInstruments, Austin, Texas, USA) and stored on a computer using data charting software (LabChart 7, ADInstruments, Colorado Springs, CO, USA).

In all groups, twitch tension was serially monitored after allowing at least a 30 min stabilization period, then T1 and TOF ratio were measured and considered as baseline values. The potency of rocuronium was tested using the cumulative dose-response method. Rocuronium (Esmeron®, MSD, Oss, The Netherlands) at an initial dose of 250 µg was added to the organ bath. Following at least 10 min of stabilization (until more than 4 twitch heights did not change) after rocuronium administration, TOF was again determined with repeated administration of 250 µg rocuronium. Subsequently, 250 µg rocuronium was added in steps following the previous dose after the least 10 min of stabilization. Thereafter, rocuronium boluses were added every consecutive 10–15 min until a complete block was achieved. When the height of T1 of the TOF was depressed under 5% of the baseline, it was considered a complete blockade. Dose-response curves were constructed and the IC50 values were measured.

To evaluate the recovery profile, 10 min after administration of the last dose of rocuronium twice the equimolar dose of sugammadex (Bridion®, MSD, Oss, The Netherlands) corresponding to the total amount of administered rocuronium was added to the organ bath. All drugs were applied using an air displacement micropipette. As the recovery profile, the following variables were measured: time to TOF ratio ≥ 0.9 as the primary outcome, the recovery index, and time to 95% of the baseline value (time of T1 recovery to 95%). (Supplementary Fig. S1).

### Tissue preparation and Immunohistochemistry

After each experiment, the hemidiaphragm muscle preparations of the rats were collected in 4% paraformaldehyde. Immunostaining was performed using the streptavidin–peroxidase method. For detection of α7-nAchRs, the muscle specimens were immediately fixed with 4% paraformaldehyde in a phosphate buffer saline and embedded in paraffin.

The endogenous peroxidase was inactivated by incubating the tissue sections in 3% hydrogen peroxide for 30 min at 25 °C. A sodium citrate buffer (0.01 M, pH 6.0, 96–98 °C) was used to recover antigens for 10 min. Then, the rabbit anti-nAChR α7 pAb (ab10096, Abcam Ltd, dilution 1:200) was added to a humidity chamber. The sections were routinely counterstained with haematoxylin. The average optical density was analysed in 5 randomly selected microscopic fields in six sections from each group at a 400-fold magnification with an optical microscope (BX51T-32F01; Olympus Corporation, Tokyo, Japan). Image analysis measurements were performed by a blind observer. The colour threshold tool of ImageJ software (ver. 1.52b; National Institutes of Health, Bethesda, MD, USA) was used to quantifying the expression of α7-nAchRs^40^.

### Evaluation of outcomes

The IC50 was the primary outcome used to depict the sensitivity to rocuronium (blockade), and the time to TOF ratio ≥ 0.9 was the primary outcome to depict reversal with sugammadex (reversal). Other recovery profiles including recovery index and time of T1 recovery to 95% were considered as the secondary outcomes for reversal. To observe the recovery period after withdrawal, both IC50 and the time to TOF ratio ≥ 0.9 were evaluated.

The experimental outcomes included: (1) effect of experimental treatment on body weight, (2) size and weight of specimens, (3) IC50 values (IC50, slope at IC50), (4) total administered rocuronium (and sugammadex) in the organ bath, (5) time to TOF ratio ≥ 0.9, (6) recovery index, (7) time of T1 recovery to 95%, and (8) average optical density of expression of α7-nAchRs.

### Statistical analysis

The competition analysis data (IC50, slope at IC50) were determined from a four-variable logistic sigmoidal dose-response model fitted to the dose-response curves using Prism 6 (Graph-Pad Software, Inc., San Diego, CA, USA). Twitch tension inhibition rates were expressed as the mean ± SEM. IC50 values (IC50, LogIC50, IC50 ratio) were expressed as means with 95% confidence intervals, while the slope at log IC50 was expressed as mean ± SEM. The IC50 ratio was defined as the IC50 of the dose-response curve in the dexamethasone group divided by that in the control group. Other data are expressed as means ± SD and were analysed with SPSS statistical software (ver. 20.0; SPSS Inc., Chicago, IL, USA).

Differences in continuous variables among the groups were analysed using analysis of variance followed by the Bonferroni method for multiple pairwise comparisons. The correlation analysis for the time to recovery and the other variables was conducted using Pearson’s tests. A two-tailed P-value of < 0.05 was considered statistically significant.

## Supplementary information

Figure S1

## Data Availability

All data are available upon request, please contact Seok Kyeong Oh (email address:nanprayboy@korea.ac.kr).
