# Supplementary material for: Effect of protracted dexamethasone exposure and its withdrawal on rocuronium-induced neuromuscular blockade and sugammadex reversal: an ex vivo rat study
Source: Sci Rep. 2019 Aug 2;9:11268. doi: 10.1038/s41598-019-47784-3 (PMC6677897; doi:10.1038/s41598-019-47784-3)
Supplement: Supplementary file 1 — Figure S1 [file 41598_2019_47784_MOESM1_ESM.pdf]

Effect of protracted dexamethasone exposure and its withdrawal on rocuronium-induced neuromuscular blockade and sugammadex reversal: an *ex vivo* rat study

Seok Kyeong Oh[1], Byung Gun Lim[1], Sungsoo Park[2], Hong Seuk Yang[3], Junyong In[4], Yong Beom Kim[5], Hey-ran Choi[6] & Il Ok Lee[1]

[1]Department of Anaesthesiology and Pain Medicine, Korea University Guro Hospital, Korea University College of Medicine, Seoul, Republic of Korea.

[2]Department of Surgery, Korea University Anam Hospital, Korea University College of Medicine, Seoul, Republic of Korea.

[3]Department of Anaesthesiology and Pain Medicine, Asan Medical Center, Seoul, Republic of Korea.

[4]Department of Anaesthesiology and Pain Medicine, Dongguk University Ilsan Hospital, Goyang, Republic of Korea.

[5]Department of Anaesthesiology and Pain Medicine, Gachon University Gil Hospital, Incheon, Republic of Korea.

[6]Department of Anaesthesiology and Pain Medicine, Inje University Seoul Paik Hospital, Seoul, Republic of Korea.

Supplementary Figure

(a)

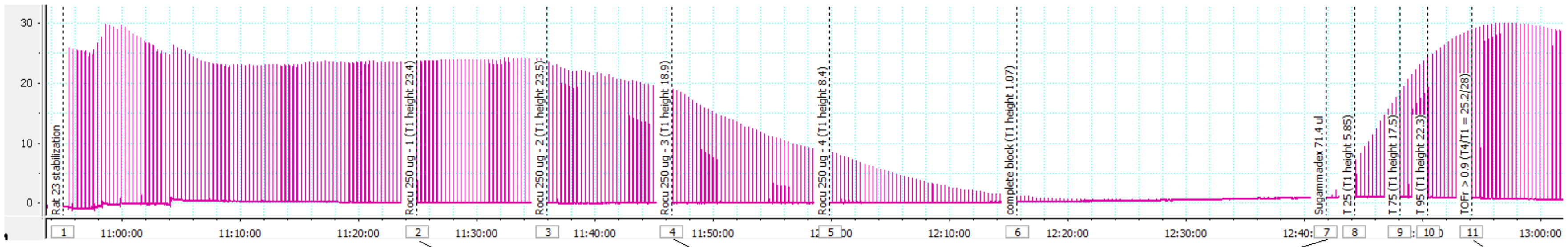

(b)

Number	Time	Comment
1	10:54:59.6944...	Rat 23 stabilization
2	11:24:57.6944...	Rocu 250 ug - 1 (T1 height 23.4)
3	11:35:59.0944...	Rocu 250 ug - 2 (T1 height 23.5)
4	11:46:30.4434...	Rocu 250 ug - 3 (T1 height 18.9)
5	11:59:54.3434...	Rocu 250 ug - 4 (T1 height 8.4)
6	12:15:44.2944...	complete block (T1 height 1.07)
7	12:41:50.6944...	Sugammadex 71.4 ul
8	12:44:14.6944...	T 25 (T1 height 5.85)
9	12:48:06.6944...	T 75 (T1 height 17.5)
10	12:50:28.8944...	T 95 (T1 height 22.3)
11	12:54:11.6944...	TOFr > 0.9 (T4/T1 = 25.2/28)

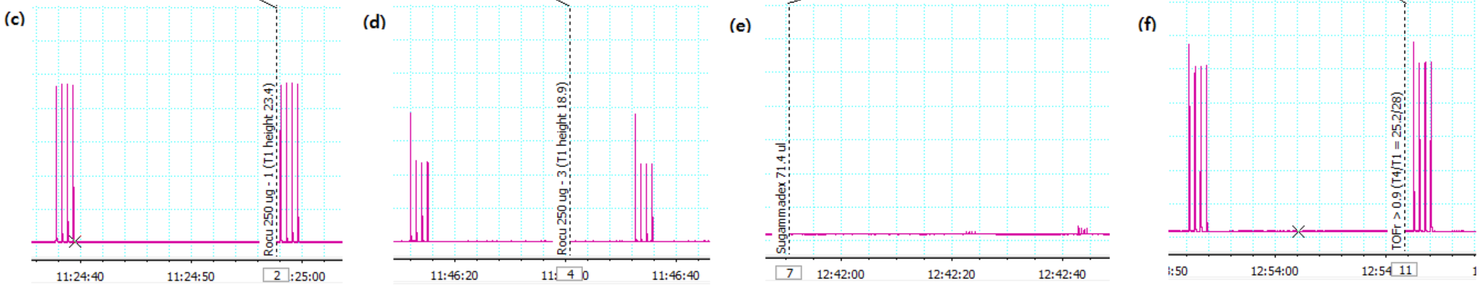

Figure S1. Representative examples of the twitch responses with the PowerLab acquisition system (ADInstruments, Austin, Texas, USA) and data charting software (LabChart 7, ADInstruments, Colorado Springs, CO, USA). (a) Time course of twitch responses during an *ex vivo* experiment. (b) The table panel showed the events in the experiment. “Number” indicates the time points. “Time” indicates the actual time. “Comment” shows the events of drug administration or measurement of the twitch response for evaluating block and reversal. (c) Train-of-Four (TOF) pattern recording at time point 2 corresponds to the first rocuronium administration. The first twitch of the TOF sequence (T1) height at this point is considered as the baseline value. (d) TOF pattern at the time point 4 corresponds to the third rocuronium administration. Decreased T1 height compared with the baseline and decreased TOF ratio from the normal ratio of 1.0 (known as ‘fade’) is shown. (e) TOF pattern at the time point 7 corresponds to sugammadex administration. Reappearance of twitch response from complete blockade is shown after sugammadex administration. (f) TOF pattern at time point 9 indicates the recovery of TOF ratio 0.9 (where T4/T1 = 25.2/28).
